# Understanding scene understanding

**DOI:** 10.3389/fpsyg.2013.00954

**Published:** 2013-12-19

**Authors:** Gregory J. Zelinsky

**Affiliations:** ^1^Departments of Psychology and Computer Science, Stony Brook UniversityStony Brook, NY, USA; ^2^Center for Interdisciplinary Research (ZiF), University of BielefeldBielefeld, Germany

**Keywords:** scene understanding, object detection, activity recognition, event detection, gist perception, eye movements, scene perception

## What does it mean to “understand” a scene?

**Figure 1:**
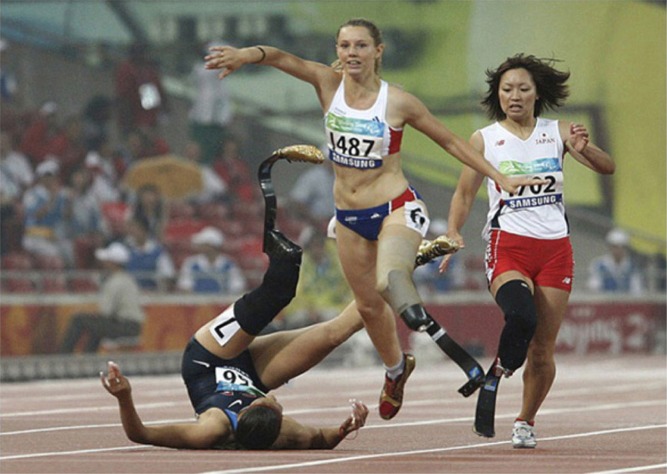
**What is this scene?**.

Look very briefly at the picture on the next page (Figure 1); what do you see? Decades of research has shown that it is possible to extract the gist of a scene very quickly (Potter, 1975; Friedman, 1979); even from the briefest of glances you could probably classify this scene as some sort of race. Other research has shown that some categories of objects, people and animals in particular, can also be detected from very brief exposures (Thorpe et al., 1996; Joubert et al., 2007). It may even be possible to infer from the features of a scene some properties of these objects, such as a person's gender and perhaps their emotion (Schyns and Oliva, 1999). Actions can also be characterized by relatively simple features (see Kozlowski and Cutting, 1977 and Wang et al., 2009, for two very different approaches), with the pose adopted by someone running being especially distinctive. So in addition to detecting that there were people in this scene, you could probably also discern that these people were women and that they were running. Combining these pieces of information might therefore have led you to a simple “gist” level of interpretation—*a women's track meet*.

But scene understanding doesn't stop at gist, and there were other things happening in this scene. Closer scrutiny reveals that one of the runners had fallen and that the others were trying to avoid tripping over her. These details may have gone undetected from a brief exposure for a variety of reasons. When a person falls their pose becomes highly variable, and certainly deviates from poses that we have associated with typical human activities. Moreover, this particular fall resulted in the loss of this person's face from view. These factors would make it harder to recognize this pattern of pixels as a person, which would obviously affect whether these details would be included in a scene interpretation following a brief exposure. “Avoidance” is also a far more difficult action to detect than running, characterized by a relatively subtle shift in body posture and a slightly exaggerating leaping stride by the runner. Had these *fallen* and *avoidance* events been detected they would almost certainly have been included in the scene interpretation, but the point is that it is unsurprising that they were not. And finally, if you had noticed that all of the runners had one prosthetic leg, this critical information would be expected to fundamentally change your understanding of this scene—suddenly it tells a story about a special race for women amputees.

This example illustrates the fact that scene understanding exists on a continuum. At one end is a very fast and seemingly effortless extraction of the scene's gist—often just its category name. At the other end is the slower and often effortful attachment of deeper meaning to the scene. Although these different interpretations likely engage different underlying processes and might therefore be given different names, for the purpose of this paper I will adopt the lay person's definition of scene understanding—what is the scene about? What is the *story* that it is trying to tell?

## Objects, actions, events, and scene understanding

Some stories are simpler than others, and for more complex tales some work is required to keep straight the various characters and how they are contributing to the story's plot. The same is true for scenes. Every story must begin with a context. Whether it is Nazi occupied France in 1942 or a simple track meet, the characters of a story must be planted in some conceptual, and sometimes just perceptual, background. This is the *gist* of a scene. Then come the characters. Whether they are black, white, or Asian female runners, animals, robots, or just inanimate things, these are the *objects* of a scene. Next is the specification of relationships between these objects, both with respect to each other and the background. Are they interacting in some way? Specifying these object relationships define the *actions* of a scene. Some actions can only be understood in the collective. In our example there were the individual actions of running and falling, but there was also the collective action of two runners avoiding the fallen runner. This is the *event* of the scene. Finally, a good story should draw a person into it. What are the characters thinking and feeling—what are their perspectives? Were the characters in our example concerned that the fallen runner might have further injured her leg, or were they focused on proving their abilities by making it first to the finish line? This is *understanding* a scene.

Is our example scene a *women's track meet or a scene about a special race for women amputees, where one runner fell and two others were trying to avoid her*? Of course the answer is that both interpretations are correct, as there cannot be one right answer. Scene understanding is ultimately an interpretation, it is whatever a person tells you it is—the specific story they have attached to a given collection of pixels. This idiosyncratic nature of scene understanding introduces unique challenges. Most obviously, if scene understanding is an interpretation, how can it be evaluated—what is the ground truth for scene understanding?

Establishing ground truth at the gist level is conceivably a tractable problem—the scene depicted in Figure 1 is clearly a *track meet* and not a *bedroom* or a *beach*. Indeed, the availability of ground truth at the gist level may be one reason why research has focused disproportionately on gist level interpretations. Ground truth for more elaborated interpretations can be obtained using a scene description task (Fei-Fei et al., 2007; Yun et al., 2013). Subjects might be asked to describe the scene that they just saw as if they were telling it to another person. Importantly, this ground truth would not be just a labeled list of the objects, actions, and events in an image (see Everingham et al., 2012). Even if each of these things could be detected with perfect accuracy—a goal that at first glance may seem desirable—the resulting list would often be an unwieldy hodgepodge of elements that would poorly approximate scene understanding. Combining semantic constraints with automated object and event detection (e.g., Farhadi et al., 2010; Yao and Fei-Fei, 2010) may successfully assemble “man,” “wall,” and “jumping” detections into a simple “man jumping over wall” interpretation—but *why* is the man jumping over the wall? It is possible to know every object and action in a scene and still not know what the scene is about—knowledge of these elements is, quite literally, not the whole story. Minimally, true understanding requires a more extensive filtering and ordering of this list to capture only those objects, actions, and events that are important to a viewer's interpretation.

## The role of eye movements in scene understanding

Scene understanding requires selecting a single interpretation from the many stories that can be made from the objects and actions depicted in a scene, and it is in this selection process that eye movements may play a role. The spatial distribution of fixations over a scene tells us the objects that a viewer finds most important. This is valuable information that might be used to exclude potentially scores of possible interpretations. This information can also be integrated with computer vision algorithms to build more intelligent image understanding systems. Object detectors corresponding to fixated locations can be biased to fire, whereas those firing at non-fixated locations can be negatively biased or removed completely. Not only would this help to avoid false positive detections (assuming imperfect detectors), it would also reduce the number of possible interpretations by pruning constructions having non-fixated subjects and objects (see Yun et al., 2013, for additional discussion and potential applications). The temporal distribution of fixations might also give clues to the object relationships. Given that subjects tend to be fixated before objects (Griffin and Bock, 2000), the ordering of fixations can help to recover the basic subject-verb-object structure that is essential to any scene understanding. With this spatial and temporal information from eye movements it may therefore be possible to distill from a morass of detected objects and events a meaningful scene interpretation—how a person is assembling a story in their mind.

The value of eye movement information might also vary with the time spent viewing a scene; shorter viewing times will lead to fewer fixations, and this in turn might interact with one's scene understanding. Without eye movements the information that can be extracted from a scene is limited to the objects and events appearing in central vision, and whatever information might be gleaned by sampling the visual periphery. This is why gist interpretations and scene categorization can be accomplished without changes in fixation, as the information enabling these minimal interpretations may be extracted globally (Oliva and Torralba, 2001; Greene and Oliva, 2009). However, the construction of detailed interpretations may require each detail to be sampled by gaze—it may not be possible to create a story about runners with prosthetic legs unless one or more of these prosthetic limbs are actually fixated during scene viewing. To the extent that this is true, it might therefore be possible to decode from fixation behavior an idiosyncratic interpretation—to predict the objects and events that a person will choose to include in their scene description (Yun et al., 2013; see also Zelinsky et al., 2013). By analyzing whether a prosthetic leg was fixated, and perhaps how often and for how long, can one predict whether this key detail will be described? Such predictions would constitute a major advance in quantifying the information used in scene understanding.

## Putting the pieces together

Gaze behavior is the thread used to weave the objects, actions, and events of a scene into the fabric of a story. This proposed role of eye movements leads to fertile theoretical ground relating scene understanding to recognition. Are eye movements made because the details of a scene tend to be visually small and relatively large in number and simply cannot be resolved and recognized without fixation (see Zelinsky, 2008, for elaboration of this view)? Or is it that these eye movements reflect the sequencing of pattern recognition events over time—the process of fitting together the pieces of the puzzle? The time taken to complete a puzzle may also depend on which pieces are placed first. To the extent that recognition is a serial process allowing for only one pattern in an image to be classified at a time, eye movements may provide a means to study the temporal unfolding of scene understanding. Sequential recognition might also inform the perceived flow of scene understanding from gist characterizations to more elaborated interpretations. Often the first pattern to be recognized may be the scene itself, the entire image of pixels. This might be followed by spatial filtering and grouping processes needed to recognize individual objects and actions. Is object recognition and scene recognition therefore one in the same? And what of the sampling of these individual recognitions, do fixations capture each or do some go unseen, and do these differences translate into different scene interpretations? Finally, it is important to ask what level lies above object recognition? Finding patterns in the relationships between objects and actions in a scene context is itself a recognition problem, one that might meaningfully be called *event recognition*. Is event recognition scene understanding, or is scene understanding something more? Moving forward it will be important to ask how scene understanding differs from an understanding of the relationships between objects and actions and events in a scene context. Certainly artists can create scenes that provoke new interpretations after hours, days, and in some cases, centuries, of viewing—should these sorts of interpretations be included in definitions of scene understanding, and if not, where should the line be drawn? Answers to these questions will shape our understanding of scene understanding.
